# Picture quiz

**Published:** 2017-02-10

**Figure:**
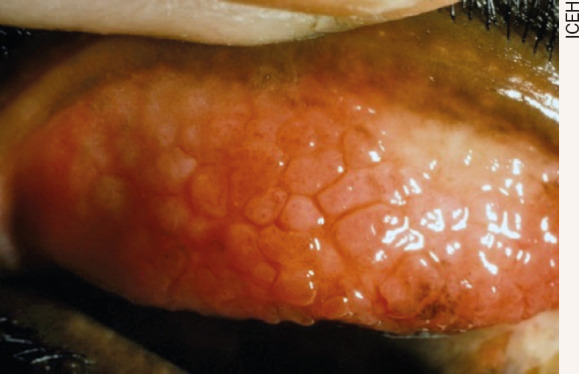


This ten-year-old boy presents with itchy, watering eyes with a thick mucous discharge of several months’ duration. His visual acuity is 6/9 and 6/12.

**Q1.** Which of the following signs are visible? (tick all that apply)
  - □ Follicles
  - □ Horner-Trantas dots
  - □ Giant papillae
  - □ Pannus
  - □ Trachomatous inflammation
**Q2.** Which of the following is the most likely diagnosis? (tick one)
  - □ Bacterial conjunctivitis
  - □ Trachoma
  - □ Kaposi's sarcoma
  - □ Vernal conjunctivitis
  - □ Adenoviral conjunctivitis
**Q3.** Which of the following may be used in treatment? (tick all that apply)
  - □ Topical prednisolone
  - □ Topical antihistamines
  - □ Topical mast cell inhibitors
  - □ Topical acyclovir
  - □ Topical neomycin

## ANSWERS

1. Answer **c.** The slide shows giant papillae (>1.0 mm) on the upper eyelid. Horner-Trantas dots may be seen on the limbus, which is not visible in this picture. There is no evidence of follicles or trachoma.
2. Answer **d.** The most likely diagnosis is vernal conjunctivitis. Bacterial conjunctivitis is associated with a purulent discharge, trachoma often shows follicles, and adenovirus is self-limiting and does not have giant papillae.
3. Answer **a, b** and **c.** Treatment is to reduce inflammation from mast cell degranulation, so mast cell inhibitors, antihistamines and prednisolone may all have a role.

